# Polyester-releasing sesamin by electrospinning technique for the application of bone tissue engineering

**DOI:** 10.1080/15685551.2022.2111857

**Published:** 2022-08-12

**Authors:** Vachira Choommongkol, Jetsada Ruangsuriya, Panawan Suttiarporn, Winita Punyodom, Boontharika Thapsukhon

**Affiliations:** a Department of Chemistry, Faculty of Science, Maejo University, Chiang Mai, Thailand; b Department of Biochemistry, Faculty of Medicine, Chiang Mai University, Chiang Mai, Thailand; c Faculty of Science, Energy and Environment, King Mongkut’s University of Technology North Bangkok, Thailand; d Polymer Research Laboratory, Department of Chemistry, Faculty of Science, Chiang Mai University, Chiang Mai, Thailand; e Department of Chemistry, School of Science, University of Phayao, Phayao, Thailand

**Keywords:** Poly(ε-caprolactone), nanofiber, electrospinning, sesamin, MG-63 cells

## Abstract

Sesamin, a significant lignin compound isolated from sesame (*Sesamum indicum Linn*), is well known for its antioxidant, anti-inflammatory, and tissue growth promotion properties. Bioabsorbable poly(ε-caprolactone) (PCL) is also a well-known polymer applied to various fields of medicine as biomaterials. The main objective of this research was to produce a prototype material from PCL and sesamin by electrospinning technique for bone tissue engineering applications. Dichloromethane and dimethylformamide (7:3) mixture was used as the solvent system for fabrication of PCL nanofiber with different loads of sesamin concentrations (1–6 wt%). The crystallinity levels decreasing and the entrapment efficiency increasing (86.87%–93.97%) were observed while sesamin concentrations were increased. The infrared spectra of electrospun mats confirmed that sesamin corporated into fibrous networks. The sesamin-loaded PCL nanofibrous membranes showed a significant release of sesamin in the range of 1.28–8.16 μg/mL within 10 weeks. The release data were fitted to zero order, first order, Higuchi and Korsmeyer-Peppas models to evaluate sesamin-releasing mechanisms and kinetics. The releasing kinetics of sesamin followed the Fickian diffusion mechanism of Korsmeyer-Peppas (R^2^ = 0.99). *In vitro* experiments with an osteosarcoma cell line (MG-63) revealed cell attachment, biocompatibility, and promotion of bone marker expression, the alkaline phosphatase (ALP) activity were studied. The electrospun PCL nanofiber loaded with sesamin had the potential as a scaffold for sesamin delivery to bone cells and applications in biomedicine.

## 1. Introduction

**Figure 1.**
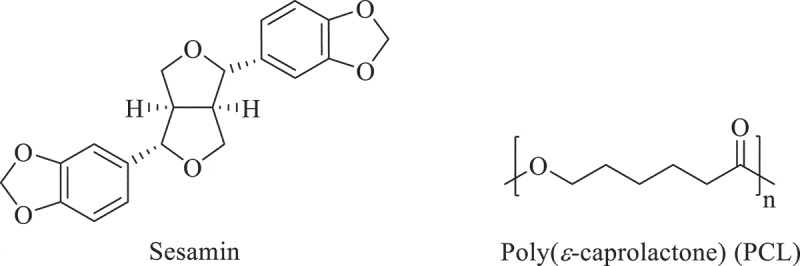
The chemical structures of sesamin and poly(*ε*-caprolactone) (PCL).

Sesamin (Figure 1) from sesame (*Sesamum indicum Linn*) is a lignan compound, widely known for its health promotion and pharmacological effects, such as antioxidant, anti-carcinogenesis, anti-hypertension, anti-dyslipidemic activities, anti-inflammation, and promotion of chondrogenesis [1,2]. In addition, sesamin promoted osteoblast differentiation in human mesenchymal stem cells [3,4,5] and chondrogenesis in the cartilage degradation model [2]. To orally deliver an effective dose of sesamin to defect organs like bone cells are obstructed by the nature of the digestive system [6] and the metabolism of sesamin by the liver [7]. Direct transportation via implanted materials is preferable for bone delivery.

Poly(*ε*-caprolactone; PCL) (Figure 1), semicrystalline, aliphatic polyester, relatively hydrophobic, consists of repeating units of five methylene groups and one ester group. PCL is currently applied in packaging, agriculture, and biomedicine due to its versatile properties such as biodegradability, biocompatibility, and an ease to blend with various polymers [8–10]. For medical and pharmaceutical applications, PCL is used in the suture, drug delivery system, and scaffold for tissue engineerings such as bone and vascular applications due to its absolute biocompatibility [11–13]. Therefore, PCL has gained much attraction to fabricate for advanced drugs delivery.

Nanofibers are a good delivery system for encapsulation with high entrapment efficiency and high surface area-to-volume ratio suitable for controlled release applications [14]. Thus, the nanofiber structure allows high permeability and cell attachment [13]. Furthermore, the encapsulation of labile molecules in nanofibers can enhance their stability under restricted biological environments. The electrospinning process is versatile and simple and has been used to produce fiber-based dressing to mimic the native extracellular matric and temporary substitute. This process involves the accumulation of charges on the surface of liquid due to the strong electrical potential to the end of a capillary containing a polymer liquid. When the voltage reaches a critical value, a charged jet ejects according to the coulombic repulsion of the charges to overcome the surface tension of the polymer droplet at the tip of the capillary. Finally, ultrafine fibers are collected on a grounded electrode due to the evaporation of the charged jet [15].

Therefore, this work aimed to produce PCL nanofiber loaded with sesamin and study the effect of different concentrations of sesamin-loaded PCL nanofibrous membranes on morphology and the release characteristics, cell attachment, biocompatibility, and alkaline phosphatase (ALP) activity on an osteosarcoma cell line (MG-63). The mathematical models for sesamin release kinetics have also been implemented according to the experimental release data. The current work is expected to provide the prototype to produce PCL nanofiber loaded with sesamin and valuable new insights into developing sesamin-loaded membranes for bone tissue engineering applications.

## 2. Experimental

### 2.1 Materials

Poly(ε-caprolactone) (PCL; ${\overset{ˉ}{M}}_{n}$ = 63,000 g/mol, inherent viscosity 1.24 dL/g in CHCl_3_) was synthesized by ring-opening bulk polymerization (ROP) at 120°C for 72 h using 0.1 mol% Sn(Oct)_2_ and 0.01 mol% n-hexanol as the initiating system. The properties of the PCL were characterized by various techniques including differential scanning calorimeter (DSC), gel permeation chromatography (GPC) and dilute-solution viscometry. Dichloromethane (DCM, RCI LabScan, 99.8%), dimethyl formamide (DMF, RCI LabScan, 99.8%) and chloroform (Carlo Erba, 99%) were used as received.

Commercial grade ethanol, ethyl acetate, dichloromethane, and hexane solvents used for extraction and purification techniques were distilled before use. Thin-layer chromatography (TLC) and column chromatography (CC) techniques were produced using silica gel 60 PF_254_ (Merck, Darmstadt, Germany).

### 2.2 Isolation of sesamin

The black sesame seeds were collected from local areas of Mae Hong Son provinces, Thailand, in 2019. Extraction and isolation of sesamin were carried out as previously described by Dar [16] and Mukhija [17] as well as their colleagues with modifications. Black sesame seeds were dried at 55°C and milled into a fine powder with grinder. 500 g powder was macerated with 2 L ethyl acetate (repeated thrice). The filtrates of the extract were combined and evaporated to obtain the crude extract (145.90 g; 29.18%). Then, 50.06 g crude extract was separated with CC using gradient ethyl acetate and hexane as mobile phase. 50 mL fraction was collected. The fractions of which *R_f_* values were similar to what sesamin presented in TLC (mobile phase; ethyl acetate:hexane (10:90) were collected. The mixed fraction was evaporated and white solid was revealed. Next, recrystallization of the solid was done with ethanol (repeated thrice) to provide sesamin crystal (0.4086 g; 0.81%).

The ^1^H and ^13^C NMR experiments were achieved by spectrometers (DRX 400 MHz, Bruker, Billerica, MA) using CDCl_3_ solvent. Chemical shifts were represented as δ-values in parts per million (ppm). Tetramethylsilane (TMS: δ 0.00) was used as internal standard. The characterization of sesamin is show as flollows: colorless crystals (EtOH), R_f_ = 0.41, mp. 120.5–122.9 °C. ^1^H-NMR (400 MHz, CDCl3): δ 3.13–2.99 (m, 2 H), 3.87 (dd, *J* = 9.3, 3.9 Hz, 2 H), 4.31–4.18 (m, 2 H), 4.71 (d, *J* = 3.9 Hz, 2 H), 5.95 (s, 4 H), 6.78 (d, *J* = 8.0 Hz, 2 H), 6.80 (dd, *J* = 8.1, 1.6 Hz, 2 H) and 6.85 (d, *J* = 1.6 Hz, 2 H). ^13^C-NMR (100 MHz, CDCl3): δ 54.31, 71.69, 85.77, 101.64, 106.48, 108.18, 118.74, 135.83, 147.09, 148.32. The spectroscopic data was congruent with previously reported data [17,18], the isolated sesamin used in this research was 98% purity and showed colorless crystals. The sesamin was used as the standard compound for developing the analytical method and loading into the PCL nanofibrous membranes for releasing and biologically testing.

### 2.3 High-performance liquid chromatography (HPLC) analysis of sesamin

280 µg/mL of sesamin was accurately prepared in methanol as a stock solution. The stock was serially diluted in methanol to generate the concentration range of 0.03–8.75 µg/mL for establishment of calibration curves. The analysis of the sesamin were carried out as previously described by Dar et al. [16] and Wang et al. [19] as well as their colleagues with some modifications. A reversed-phase Brownlee C18 column (250 mm, 4.6 mm, 5 µm, Perkin Elmer, Waltham, MA, USA) at 35 ± 1 °C was used with freshly prepared acetonitrile and water containing 0.1% v/v phosphoric acid mixture (60:40, v/v) as a mobile phase. HPLC-graded acetonitrile and water were purchased from RCI labscan (Bangkok, Thailand). The HPLC system consisted of a PerkinElmer Flexar^tm^ HPLC equipped with photodiode array detector (PAD) detector (PerkinElmer, Waltham, MA, USA). Data were collected and processed with the PerkinElmer Chromera CDS Software. All solutions were filtered separately through a 0.22 μm nylon membrane filter before use. The flow rate of the mobile phase was set at 1.0 mL/min. A sample volume of 15 μL was injected, and the wavelength of 290 nm was used to detect sesamin in the eluent with 10 min total run time. Sesamin contents were derived from the average of the triplicate results in each sample.

### 2.4 Fabrication of sesamin-loaded PCL nanofiber membranes

20% (w/v) PCL solution was prepared by dissolving the PCL in 7:3 (v/v) DCM:DMF. Then, different sesamin amounts were added to the solution to generate sesamin-loaded PCL solutions at different concentrations (1–6 wt%) designated as 1%SM-PCL – 6%SM-PCL while PCL (0%SM-PCL) was a control without sesamin. All the solutions were magnetically stirred at room temperature for 6 hours and placed into a syringe connected to a stainless-steel blunt-ended needle 22-gauge with an 0.8 mm. outer diameter. A voltage of 20 kV was set during electrospinning, and the distance between the nozzle and the grounded collector was 18 cm. A whole series of sample membranes with or without sesamin loaded were fabricated 3 hours without an interval, resulting in the thickness of the fiber mats of 150 ± 15 μm. All the membranes were further dried for 24 hours at ambient temperature in a vacuum drying oven to remove organic solvent residues.

### 2.5 Characterization of sesamin-loaded PCL nanofiber membranes

The Fourier transform infrared spectrophotometry (FTIR) transmission spectra were performed within the spectral range of 4,000–400 cm^−1^ on a Fourier transform infrared spectrophotometer (Nicolet iS5, Thermo Scientific) through the accumulation of 32 scans with a resolution of 4 cm^−1^, in attenuated total reflectance spectroscopy (ATR) mode. The morphologies of the PCL nanofiber membranes were characterized by scanning electron microscopy (SEM, Quanta 250, FEI). The nanofiber membranes were coated with gold before observed at a voltage of 15 kV. The average fiber diameter and its distribution were determined on SEM micrographs at 100 random locations using ImageJ software. Differential scanning calorimetry (DSC) was performed to investigate the thermal properties of the membranes. DSC analyses were carried out in a Mettler-Toledo DSC3 instrument with STARe Default DB V12.10-STARe software under a nitrogen flow rate of 20 mL/min and heating rate of 10 °C/min from 0 to 200 °C. Gel permeation chromatography (GPC) analyses were carried out on Waters 2414 refractive index (RI) detector, equipped with Styragel HR5E 7.8 × 300 mm column (MW resolving range = 2,000–4,000,000). The GPC columns were eluted with a tetrahydrofuran flow rate of 1.0 mL/min at 40 °C and calibrated with polystyrene standards.

### 2.6 In vitro *hydrolytic degradation*

The electrospun SM-PCL membranes were cut into circular form with 10 mm in diameter for *in vitro* hydrolytic degradation studies. A pre-weighed membrane from each condition was placed into a 2 mL Eppendorf tube containing 1 mL phosphate buffer saline (PBS, pH 7.40) and incubated at 37.0 °C for 10 weeks. After that, the sample was collected, washed with deionized water, and dried in a vacuum oven at RT to constant weight. GPC was used to determine the molecular weight reduction of the SM-PCL in respect of time. Furthermore, the pH of the solution was also determined.

### 2.7 In vitro *release of sesamin-loaded PCL nanofiber membranes*

#### 2.7.1 Entrapment efficiency

All SM-PCL samples were completely dissolved in CHCl_3_ and methanol (1:9 v/v) before determining the sesamin by HPLC. Entrapment efficiency was calculated by the amount of sesamin determined by HPLC and by theoretical determination as follows:

$$\begin{array}{rl} & Entrapment\\ & \;\;\;efficiency\left(\%\right)=\frac{weight\;of\;sesamin\;in\;the\;membrane}{\begin{array}{c} theoretical\;weight\;of\;sesamin\\ \;\;\;\;\;\;\;\;\;\;\;\;\;\;\;\;\;\;\;\;\;\;loaded\;in\;the\;membrane \end{array}}\times 100\% \end{array}$$

#### 2.7.2 Sesamin release assay

The releasing profile of sesamin in the SM-PCL was established similarly to *in vitro* hydrolytic degradation. In addition, 1 mL of the mixture were drawn from each time point and filtered through a 0.45 µm Nylon membrane for HPLC analysis of the sesamin released. Subsequently, 1 mL of the fresh PBS was replaced with the drawn solution into each condition. Releasing of sesamin was observed everyday for 7 days and every week for 10 weeks. The cumulative sesamin released was detected by HPLC. The absorption peak area of sesamin was converted to the concentration of drugs through a calibration curve of sesamin in the same buffer solution.

### 2.8 Release kinetics

The sesamin release data were kinetically evaluated by zero-order, first-order, Higuchi, and Korsmeyer-Peppas models. After estimating the regression coefficient value (R^2^), the best kinetic model was obtained.

## Zero-order model

This model has also been used to slowly describe drug release and dissolution from dosage forms that do not disaggregate. The equation can represent the release:

(1) $$Q_{t}=Q_{0}+k_{0}t$$

where $Q_{t}$ is the amount of drug dissolved in time t, $Q_{0}$ is the initial amount of drug in the solution (most times, $Q_{0}=0$) and $k_{0}$ is the zero-order release constant expressed in a unit of concentration/time.

## First order model

The equation can express the release of the drug that followed first order:

(2) $$logQ_{t}=logQ_{0}+\frac{k_{1}t}{2.303}$$

where $k_{1}$ is the first order constant, equation (2) corresponds to a linear function, and the graph of the logarithm of the drug release will result in a straight, with angular coefficient $K_{1}/2.303$ and linear coefficient equal to $logQ_{0}$.

## Higuchi model

The simplified Higuchi model can be used to determine the drug release from modified release dosage forms such as some transdermal systems that related the concentration of drug to the square root of time:

(3) $$Q_{t}=K_{H}\sqrt{t}$$

where $K_{H}$ is the release constant of Higuchi

## Korsmeyer-Peppas model

This model was developed by Korsmeyer *et al*. [20] to release a drug molecule from a polymeric matrix. The drug release mechanisms are based on drug diffusion, polymer swelling, and erosion [21]. The semi-empirical equation to describe drug release from the polymeric system is the so-call power law:

(4) $$\frac{M_{t}}{M_{\infty }}=kt^{n}$$

Here, $M_{t}$ and $M_{\infty }$ are the absolute cumulative amount of drug release at time t and infinite time, respectively, k is a kinetic constant incorporating structural and geometric characteristics of the device, n is the release exponent, indicative of the mechanism of drug release. The release mechanism; for n < 0.5, a pseudo-Fickian diffusion mechanism; n = 0.5, a Fickian diffusion mechanism; 0.5 < n < 1, an anomalous diffusion mechanism; and for n = 1, a non-Fickian diffusion mechanism controls the mechanism release.

### 2.9 Cellular activity of sesamin-loaded PCL nanofiber membranes

#### 2.9.1 Material preparation

The SM-PCL nanofiber membranes were prepared on the surface of 10 mm diameter aluminum foil discs and placed onto the surface of a 24-well plate with a cosmetic grade petroleum jelly (Vaseline) aid for fixing. The disc was subjected for a 30-minute UV sterilization prior to cell loading.

#### 2.9.2 Cell culture

MG-63 cell line (ATCC, CRL1427), an osteosarcoma cell, was evaluated for cell attachment and biocompatibility of the polymer. The cells were cultured in a complete Dubecco’s modified Eagle’s medium (DMEM) containing 10% fetal bovine serum (FBS), 2%HEPES, 1% non-essential amino acid, and 1% penicillin/streptomycin until they reached confluence. The cell densities of 2.5 ×10^5^ cells/mL and 1 ×10^5^ cells/mL were prepared for cell attachment and biocompatibility, respectively. 1 mL of the cell suspension was loaded into each well containing the SM-PCL.

#### 2.9.3 MTT assay for cell attachment, viability, and proliferation

Attachment of MG-63 cells on SM-PCL was observed at 6, 12, and 24 hours in a 37 °C, 5% CO_2_ humidified incubator while viability and proliferation were observed at 4 and 7 days. When due,

100 μL of 5 mg/mL methylthiazolyldiphenyl-tetrazolium bromide (MTT) (Sigma, M2128) solution in PBS pH 7.4 (Gibco, 10,010,023) was added into each well. The plate was continued incubated for 4 hours. 1 mL DMSO (VWR, 0231) was used to solubilize the formazan crystals on the SM-PCL discs. The solubilized solution was read the absorbance at 540 nm with a plate reader (BioTek, SynergyH4) and the cell numbers were calculated from the standard curve.

#### 2.9.4 Biocompatibility tests

When the cells on SM-PCL were due on 4 and 7 days, the cell lysate was collected using 300 μL cell lysis buffer (Sigma, C2978). The lysate was collected into an Eppendorf tube and stored at −20°C for further analyses.

##### 2.9.4.1 Bradford assay for total protein determination

The Bradford assay was done by mixing 10 μL of the lysate with 200 μL of the Bradford solution (AppliChem, A6932,0100). The mixture was allowed at room temperature for 15 minutes and the absorbance was measured with the plate reader at 595 nm. The protein concentrations were calculated according to the bovine serum albumin (BSA) (BioBasicCanada, D0023) standard curve.

##### 2.9.4.2 Alkaline phosphatase (ALP) activity determination

The ALP activity was determined by mixing 20 μL of the lysate with 80 μL of 1 mg/mL *p*-nitophenolphosphate (pNPP) (Sigma, N2640), as a substrate, in diethanolamine (Sigma, D8885) buffer pH 9.8. The reaction was incubated at 37 °C for 3 hours. The absorbance of the mixture was measured at 405 nm with the plate reader, and the activity was calculated using standard *p*-nitophenol (pNP) (Sigma, 1048). Normalization of the activity with the total amount of the protein in the reaction was reported as a specific activity.

### 2.10 Statistical analysis

The results were illustrated as mean ± SD from a representative of a triplicate experiment. The comparison among SM-PCL at different concentrations was performed with ANOVA followed by Tukey *pos-hoc* test of which the significant level was set at p < 0.05.

## 3. Results and discussion

### 3.1. HPLC analysis and method validation

The linearity, limit of detection (LOD), limit of quantification (LOQ), precision and accuracy for the quantification of analytes were validated. The method revealed good linearity in the range of sesamin concentrations of 0.03 to 8.75 μg/mL. Peak identification was made by comparison with retention times (RT) at 5.39 min. The analytical curve generated a linear equation (y = 17419x + 147.5) with high correlation coefficient (R^2^ = 0.9998). The LOD and LOQ values for the analytes were 0.07 μg/mL and 0.22 μg/mL, respectively. The precision data were conducted by five repetitive injections on the same day and consecutive 3 days. The RSD value for intra-day and inter-day precisions were 1.43% and 1.52%, respectively. The quantification procedure was precise, as indicated by the low RSD value (less than 5%) recommended by ANVISA [22]. The mean recovery results of sesamin were 107.94% with RSD 1.58%, showing that the developed method was reproducible and had sufficient accuracy.

### 3.2 Fabrication and characterization of sesamin-loaded PCL nanofiber membranes

PCL was dissolved immediately in DCM and was taken a little time in DMF. As known, if the applied voltage is not high enough, the electric force can not overcome the surface tension. However, the spinnability of a polymer solution is better if the conductivity is high. DMF and DCM have conductivity values about 1.59 ×10^−5^ and 4.31 ×10^−9^ S/m, respectively [23]. For this reason, DMF was chosen to use as a mixed solvent with DCM to prepare the PCL solution.

**Figure 2.**
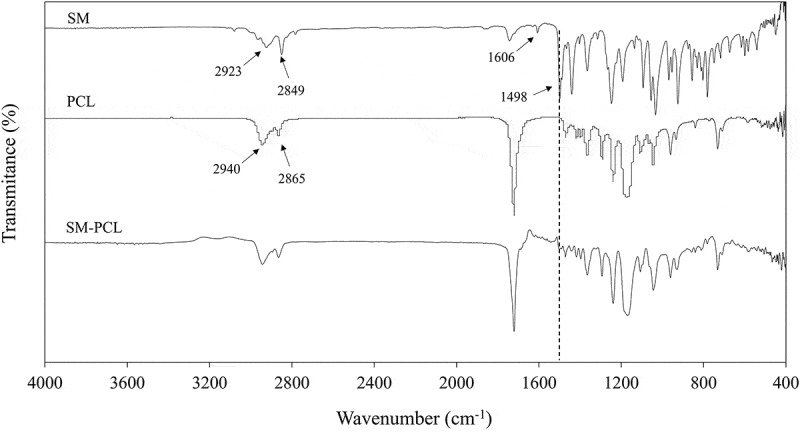
FTIR spectra of sesamin (SM), nanofibrous PCL membrane without loaded sesamin (PCL) and seamin-loaded PCL (SM-PCL) membrane.

The characteristic FTIR peaks of sesamin (SM), PCL nanofibrous membranes without sesamin (PCL) and PCL nanofibrous membranes with sesamin loading (SM-PCL) are shown in Figure 2. The FTIR spectrum of sesamin signals can be observed at 3085, 2923, 2849 cm^−1^ corresponding to =C-H and C-H stretching. The absorption band of C =C stretching appeared at 1606, and 1498 cm^−1^ related to a benzene ring. The spectrum of sesamin was in agreement with previously reported data [17].

The FTIR spectrum of PCL signals can be observed at 2944 and 2865 cm^−1^ corresponding to asymmetric and symmetric of the methylene-oxygen (CH_2_-O), respectively. The strong signal at 1720 cm^−1^ corresponds to the carbonyl bonds (C =O) and belonging to the aliphatic ester (CO_2_-) of the PCL. Moreover, the asymmetric and symmetric C-O-C bands peaked at 1239 and 1170 cm^−1^, respectively [24].

The FTIR spectrum of SM-PCL showed the main stretching and bending vibration similar to the PCL polymer. This may due to small sesamine amount at the surface of the membrane because it is uniformly distributed across the membrane and presented in a low concentration in the blend. Furthermore, the formation of electrospun SM-PCL could be confirmed by the weak absorption bands at 1498 cm^−1^ of the C =C stretching vibrations of the sesamin.

### 3.3 Morphology of the PCL membranes

**Figure 3.**
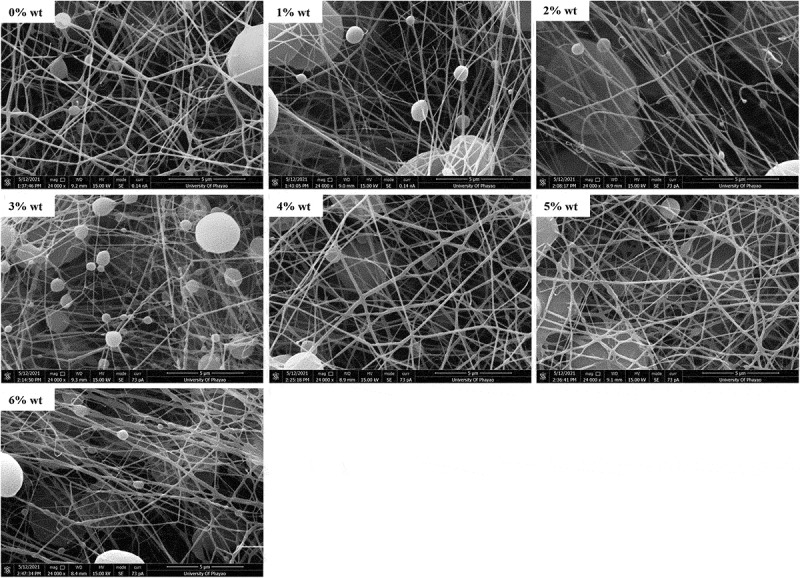
SEM images of PCL nanofibrous membranes. The PCL without sesamin loaded (0%SM-PCL) and sesamin-loaded PCL (SM-PCL) nanofibrous membrane with different concentrations of loaded sesamin at 1–6 wt% (1%SM-PCL – 6%SM-PCL).

Nanofibrous membranes were revealed by SEM in a bead-on-string fashion (Figure 3). PCL nanofibrous membrane without loaded sesamin showed a uniformly continuous stable fiber with minimal bead-on-string structure. Saberin et al. prepared PCL electrospun nanofibers decorated with copper hexacyanoferrate as an ion exchanger for effective cesium ion removal. The SEM images of the PCL nanofibers have a homogeneous fibrous structure with the diameter of the electrospun nanofibers ranged from 350 to 500 nm. It is observed clearly that the fibers are cross-sectionally round and their surfaces are smooth [25]. Moreover, Mirzaeei et al. also fabricated PCL nanofibers containing metronidazole and amoxicillin for the treatment of periodontitis. Scanning electron microscopy showed that the drugs containing PCL nanofibers was uniform and bead-free in morphology with the mean fiber diameter as 282 ± 68 nm [26]. Likewise, the bead-on-string structure was more prominent when sesamin concentration was increased without any alteration of fiber diameter.

The average fiber diameters varied from 110 to 140 nm as well as the size of the bead varied from 2.6 to 3.3 mm, which were not significantly different between sesamin presence and absence (Table 1). It is known that the constant distance between the needle and the collector and the constant breakdown of the jet flow to droplets can occur due to low molecular weight or low concentration of polymer solution [13]. In the case of bead-on-string formation, it allows entrapment of particle drugs inside the beads. Thus, the bead morphology is somehow more important than the diameter of the nanofibers because the beads can encapsulate some portion of sesamin or other drugs for delivery applications. According to the results of Li et al. [27], the ratio of bead length (BL) to bead diameter (BD) contained the information of the uniformities of nanofibers and beads diameter was determined how well drugs molecules could be entrapped inside the beads. Moreover, the beaded nanofiber morphology was changed with polymer concentration and solvent ratio. The lower the polymer solution concentration, the smaller the diameter bead could yield, in vice versa. The BD distribution of sesamin-loaded beaded nanofibers was not significant with various sesamin concentrations in this work. That is the foundation for further evaluating the effect of sesamin on the release profiles of beaded nanofibers.

**Table 1.** Fiber diameter, bead diameter, and %crystallinity of sesamin loaded PCL membranes (n = 3).

Theoretical sesamin loading (%wt)	Fiber diameter (nm)	Bead diameter (μm)	% crystallinity^a^
0	132 ± 23	2.64 ± 1.1	10.4
1	114 ± 38	3.26 ± 2.0	n.d.
2	131 ± 39	3.04 ± 2.3	n.d.
3	116 ± 32	2.62 ± 1.7	10.2
4	140 ± 38	2.46 ± 1.4	10.1
5	132 ± 28	2.49 ± 1.2	9.5
6	123 ± 41	2.77 ± 1.7	9.1

^a^these values calculated from DSC with the enthalpy energy of 100% crystal PCL 139.5 J/g

The %crystallinity from DSC results of electrospun PCL and SM-PCL membranes are shown in Table 1. Adding sesamin to the solution, the sesamin molecules distributed in the semi-crystalline PCL matrix caused a tendency reduction in crystallinity of PCL. While solvent evaporated in the electrospinning process, sesamin may be inserted between PCL chains and interrupt the chain orders.

### 3.4 In vitro *hydrolytic degradation*

**Figure 4.**
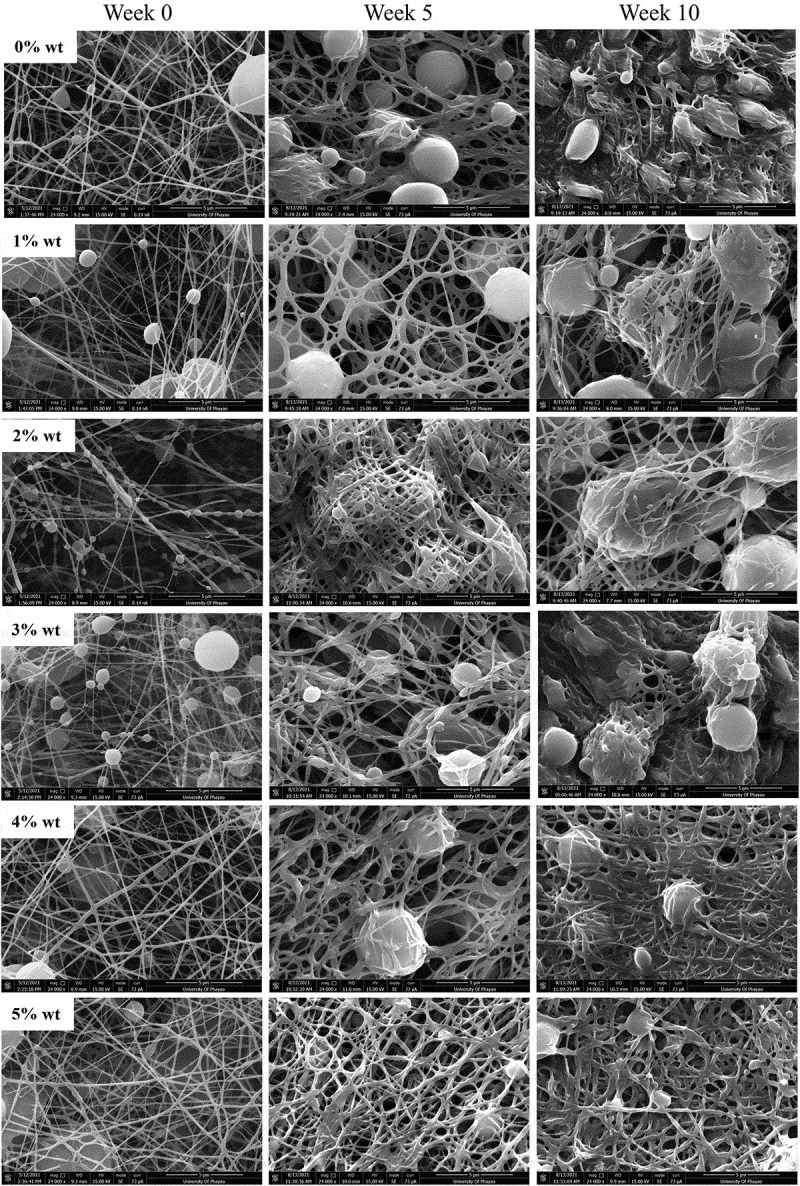
SEM images of sesamin-loaded PCL nanofibrous membranes subjected for *in vitro* hydrolysis degradation. The fibers were taken with SEM before submerge (week 0) and submerged for 5 and 10 weeks. The PCL without sesamin loaded (0%SM-PCL) and sesamin-loaded PCL (SM-PCL) nanofibrous membrane with different concentrations of loaded sesamin at 1–5 wt% (1%SM-PCL – 5%SM-PCL).

PCL or 0%SM-PCL and SM-PCL were spontaneously degraded after being submerged in PBS at 37 °C for 10 weeks. Significant changes in morphology were observed in PCL or 0%SM-PCL and SM-PCL after *in vitro* hydrolytic degradation (Figure 4). The changes were clearly observed from 5 weeks of incubation, illustrating the swell of the electrospun nanofibers and merging fibers were highlighted at 10 weeks of degradation by the loss of nanofiber pattern, and the sesamin loaded PCL beads were revealed. However, the concentrations of sesamin in SM-PCL were not significantly different on nanofiber degradation patterns due to the insignificant difference in fiber diameter and bead size.

The decreases in ${\overset{ˉ}{M}}_{n}$, ${\overset{ˉ}{M}}_{w}$ and polydispersity (PD) of PCL and SM-PCL membranes with degradation time were presented in Table 2. The initial reductions are typical of a random chain scission mechanism. Despite being more easily hydrophobic, the electrospun PCL membranes are semicrystalline. Thus, hydrolysis occurs in the amorphous regions of the matrix where the chains are more loosely packed than in the highly ordered crystalline parts, and that diffusing water molecules could access the hydrolyzable ester bonds. From our previous work, the electrospun poly(L-lactide-co-caprolactone) copolymer with high caprolactone unit PLC 50:50 was slower the rate decrease of ${\overset{ˉ}{M}}_{n}$ than that of the PLC 67:33 [28]. The ${\overset{ˉ}{M}}_{n}$ of PCL gradually decreased more than SM-PCL due to the naturally hydrophobic of sesamin particles, and it may disrupt the transportation of water molecules.

**Table 2.** Molecular weight loss and polydispersity (PD) of PCL nanofibrous membrane without sesamin loaded (0%SM-PCL) and sesamin-loaded PCL (4%SM-PCL) (n = 3).

Sample	Degradation time (weeks)	Average molecular weight		PD
		${\bar{M}}_{n}$	${\bar{M}}_{w}$	
0%SM-PCL	0	18,292	31,364	1.67
	5	18,090	30,751	1.70
	10	16,675	27,855	1.71
4%SM-PCL	0	19,726	32,369	1.64
	5	18,917	31,398	1.66
	10	17,672	29,344	1.66

**Figure 5.**
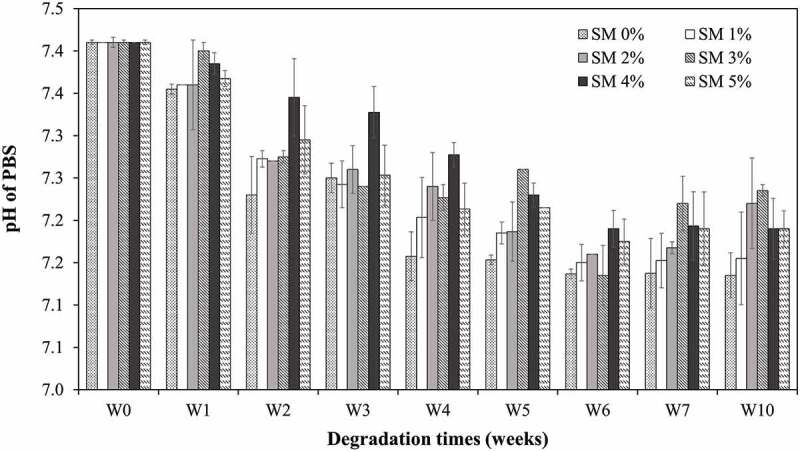
pH values profile of PCL nanofiber dissolution for 10 weeks. The PCL without sesamin loaded (0%SM-PCL) and sesamin-loaded PCL (SM-PCL) nanofibrous membrane with different concentrations of loaded sesamin at 1–5 wt% (1%SM-PCL – 5%SM-PCL).

Due to the hydrophobicity of PCL, the degradation proceeds via surface erosion rather than bulk erosion. Finally, this surface erosion, combined with the continued molecular weight decrease, causes porous structures to be destroyed. Thus, the water molecules access the bulk interior of the polymer matrix by accelerating the degradation. Simultaneously, the decreasing pH (Figure 5) of the PBS solution, resulting from the release of acidic hydrolysis products and contributes to accelerating the degradation by acid-catalysis of the ester hydrolysis mechanism.

### 3.5 Entrapment efficiency

The entrapment efficiency of sesamin-loaded PCL slightly increased when the sesamin concentration increased from 1 to 5 wt%, whereas it slightly dropped when sesamin was 6 wt%. The percent entrapment values were at 86.87 ± 5.1, 92.13 ± 8.3, 93.71 ± 7.8, 97.81 ± 4.2, 97.91 ± 5.2, and 93.97 ± 6.4 of 1%SM-PCL to 6%SM-PCL, respectively. Sesamin at a high concentration tended to locate on the nanofiber surface. The sesamin and PCL compatibility is necessary for a perfect encapsulation of the sesamin inside the electrospun PCL nanofibers during the rapid solvent evaporation and quick stretching in the electrospinning process.

### 3.6 Release of sesamin

**Figure 6.**
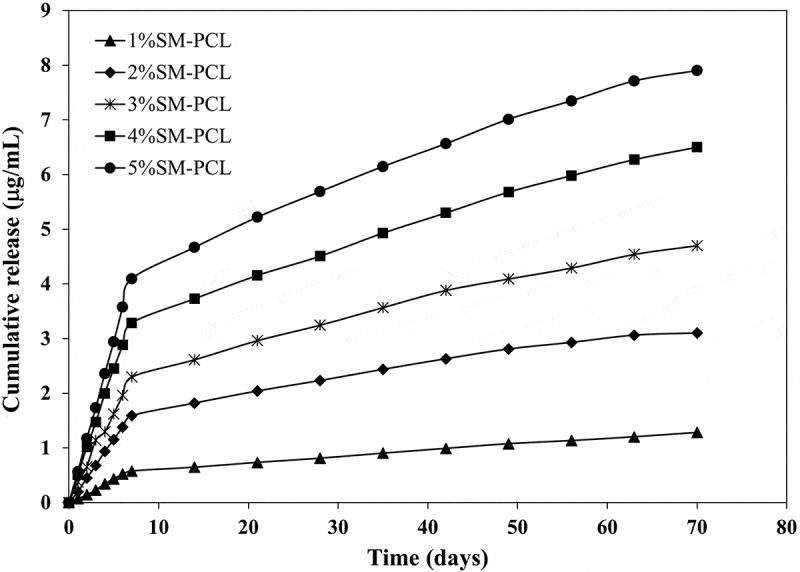
Sesamin release profile of PCL nanofiber loaded with different concentrations of loaded sesamin at 1–5 wt% (1%SM-PCL – 5%SM-PCL).

Releasing sesamin from the SM-PCL nanofibrous membrane into the aqueous solution showed a similar trend of release when the concentrations of the sesamin were different (Figure 6). Increasing sesamin concentration from 1% to 5% wt significantly affected the release rate. However, the rate was constant when the sesamin concentration was 5% wt or over. The sesamin contents of 1.28 ± 0.03, 3.21 ± 0.05, 4.82 ± 0.42, 6.63 ± 0.25 and 8.16 ± 0.38 μg/mL were released from 1%SM-PCL – 5%SM-PCL within 10 weeks of study, respectively. It is believed that at higher concentrations of sesamin, the sesamin in the polymer solution had more inclination to migrate to near the surface of nanofibers during the electrospinning process. Thus, the exposure and diffusion of sesamin to the PBS solution became higher, leading to a faster sesamin release rate in the first period (7 days). The other reason may be related to PCL nanofibrous membranes’ physical and structural properties. From DSC results, increasing the amount of sesamin in PCL nanofibrous membranes caused a tendency to reduce PCL crystallinity (Table 1). Drugs may be incorporated into the amorphous regions of semicrystalline polymers.

As illustrated in Figure 6, the sesamin release continued for at least 10 weeks. This was the first study of the sesamin release system from PCL nanofibers and successful encapsulation of sesamin. The PCL nanofibers were the leading cause of prolonged drugs release and low burst release [29]. It can be seen that bead-on-string nanofibers did not significantly affect sesamin release profiles. The rate of sesamin releases depended on the amount of sesamin encapsulated in nanofibers. After 7 days, the sesamin release rate slowed down until accumulated sesamin release became almost steady. Moreover, the 2%SM-PCL – 5%SM-PCL release rate become plateau after 63 days. The SM-PCL membranes may become good carrier candidates to be employed in a range of bone applications with the requirement of slow drug release.

### 3.7 Release kinetic

The release kinetic of sesamin is determined by fitting four aforementioned mathematical models to the release data. The calculated kinetic values are presented in Table 3, and the release data were more fitted with the Korsmeyer-Peppas model with regression coefficient values (R^2^) higher than 0.9862.

**Table 3.** Fiber diameter, bead diameter, and %crystallinity of sesamin loaded PCL membranes (n = 3).

Theoretical sesamin loading (%wt)	Fiber diameter (nm)	Bead diameter (μm)	% crystallinity^a^
0	132 ± 23	2.64 ± 1.1	10.4
1	114 ± 38	3.26 ± 2.0	n.d.
2	131 ± 39	3.04 ± 2.3	n.d.
3	116 ± 32	2.62 ± 1.7	10.2
4	140 ± 38	2.46 ± 1.4	10.1
5	132 ± 28	2.49 ± 1.2	9.5
6	123 ± 41	2.77 ± 1.7	9.1

^a^these values calculated from DSC with the enthalpy energy of 100% crystal PCL 139.5 J/g

The Korsmeyer-Peppas model was explicitly developed to release a drug molecule from a polymeric matrix [30]. It was a good agreement with the polymeric system in this study. Moreover, the diffusion coefficient values (n) on all nanofibrous membranes were n < 0.45, confirming the sesamin release mechanism following Fickian diffusion and the structure of the beads-on-a-string membranes had no significant effect on the sesamin diffusion mechanism. Thus, Fickian diffusion is a mechanism explaining that molar flux is proportional to its concentration gradient. Similar to Fickian diffusion, the diffusion mechanism of Korsmeyer-Peppas also occurs due to the swelling effect of polymer matrixes. When the sesamin concentration increases from 1 to 5 wt%, the kinetic constant also increases from 0.1864 to 1.4393, suggesting the increase of sesamin release and diffusivity due to the greater amount of free sesamin molecules on the PCL nanofiber surfaces.

Therefore, the release data can be used to predict the efficacy of drug release system and can help in treatments. The release behavior of chloramphenicol as an antibacterial drug from PCL nanofibers has been investigated by Arbade, G.K. and coworkers [31]. The release profile of PCL-chloramphenicol electrospun nanofiber was fitted with the Ritger–Peppas and Korsmeyer–Peppas model. The power-law exponent, n was found to be between 0.36 and 0.72. The scaffold showed the n value of 0.36, implying the drug release by Fickian diffusion (n > 0.5). However, the scaffolds with higher amounts of drug showed the n values > 0.5, indicating the drug release by non-Fickian diffusion. This may be related to the smaller diameter of nanofibers resulting in higher surface area. Moreover, Armedya et al. also studied naproxen’s kinetic release from copper ferrite nanoparticles incorporated on PCL/collagen nanofiber. They reported that the release profile was fitted to the Korsmeyer-Peppas model and the release mechanism was a combination of Fickian diffusion and swelling [32]. Furthermore, Huo et al. prepared PCL/collagen nanofibers containing artemisinin (ATR) for transdermal drug delivery system that showed the release of ART from the PCL/Col nanofibers followed the Korsmeyer-Peppas model. The diffusion exponent value (n) from the fitting data of ART released from PCL/Col was in range of 0.40–0.48, indicating that ART release is controlled by diffusion throughout the release process [33]. Based on the above investigation, it is hypothesized that the release process of sesamin from sesamin loading PCL nanofibrous membrane presents the following mechanism. First, PCL nanofibrous membrane could absorb water and swell. Subsequently, sesamin molecules diffuse into the PBS medium from the surface of the PCL nanofibers. The swelling of nanofibers leads to cleavage of interaction between sesamin and PCL nanofibers that helps to release and diffuse sesamin from nanofibers. The medium gradually penetrates the nanofibers as the release progresses. Then, the sesamin embedded in the PCL nanofibers gradually dissolves and eventually completely dissolves in the medium [33,34].

### 3.8 Attachment of MG-63 cells on PCL and SM-PCL

**Figure 7.**
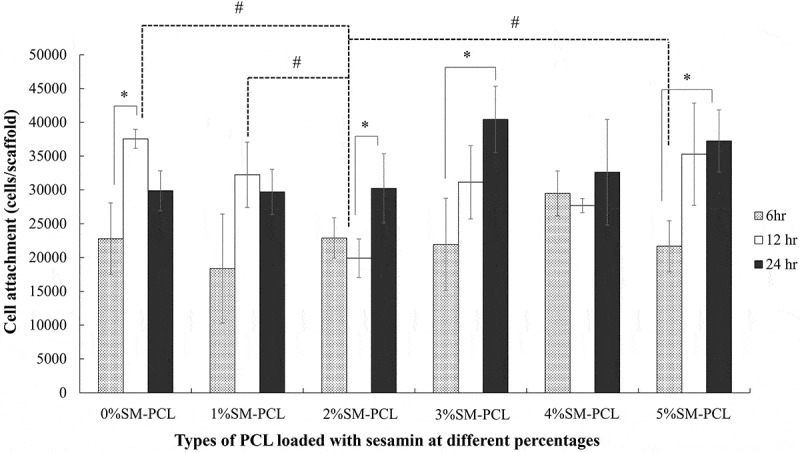
MG-63 cell attachment to polycaprolactone (PCL) or sesamin-loaded PCL (SM-PCL). MTT assay of MG-63 cells attached to PCL or SM-PCL with concentrations of 1–5% (1%SM-PCL – 5%SM-PCL) for 6 hr, 12 hr, and 24 hr. * indicates the significant difference among three periods of incubation within the same types of the tested material while # indicates the significant difference among types of tested materials within the same period with n =3; p < 0.05.

**Figure 8.**
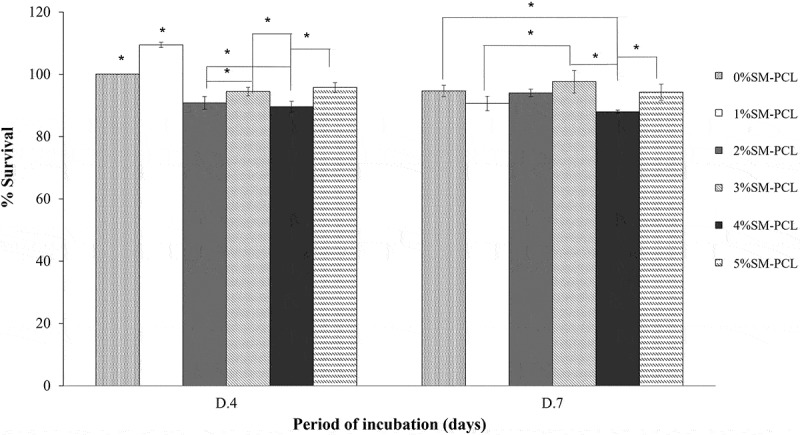
MG-63 cell viability and proliferation. MTT assay of MG-63 cells cultured with polycaprolactone (PCL) or sesamin-loaded PCL (SM-PCL) with concentrations of 1–5% (1%SM-PCL – 5%SM-PCL) for 4 (D.4) and 7 days (D.7). * indicates the significant difference among types of tested materials within the same period with n =3; p < 0.05.

The presence or absence of sesamin in PCL did not alter the ability of the MG-63 cells to attach to the polymer. There was no significant number of cells attached to the polymer with or without sesamin after 6 and 24 hours. However, 2%SM-PCL resulted in a significant reduction in cell attachment compared with the control 0%SM-PCL and 1–5%SM-PCL. Moreover, the number of cells attached to the materials was significantly affected by time (Figure 7). As illustrated in Figure 8, the significant increases in cell number with time were observed in 0%SM-PCL, 2%SM-PCL, 3%SM-PCL, and 5%SM-PCL between time points of 6 and 12 hours, 12 and 24 hours, 6 and 24 hours, and 6 and 24 hours, respectively. These results supported the ability of the PCL and SM-PCL to allow the MG-63 attachment as a critical step for cell growth.

### 3.9 Cell viability and proliferation of MG-63 cells on PCL and SM-PCL

The presence or absence of sesamin in PCL did not activate cell proliferation of MG-63 from 4 to 7 days of culture. Even though there were significant differences in viability percentage among tested samples within the same incubation period, the viability percentage of the cells cultures on the same materials between 4 and 7 days showed insignificant viability. This result indicated the cells tend to stop proliferation after 4 days of culture, and there was no toxicity of the tested material to the MG-63 cells as present in Figure 8. The result could imply the biocompatibility of the PCL and SM-PCL in all of the concentration ranges.

### 3.10 Total protein production

**Figure 9.**
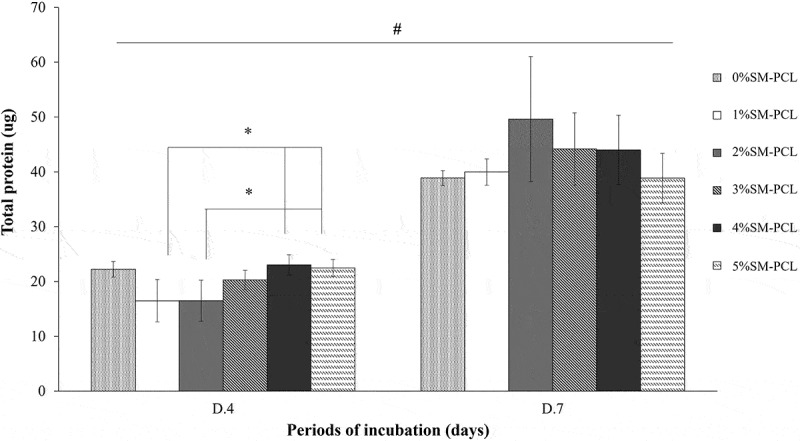
Total protein production of MG-63. Bradford assay for total protein determination of MG-63 cells cultured with polycaprolactone (PCL) or sesamin-loaded PCL (SM-PCL) with concentrations of 1–5% (1%SM-PCL – 5%SM-PCL) for 4 (D.4) and 7 days (D.7). * indicates the significant difference among types of tested materials within the same period while # indicates the significant difference between two incubation periods within the same types of the tested material with n =3; p < 0.05.

The presence of sesamin did not affect total protein production in MG-63 cells. Sesamin at a low percentage (1% and 2%) caused a significant reduction in total protein reduction in 4 days of culture, while the higher percentage (4% and 5%) caused restored the total level of protein. In contrast, total protein levels were similar in all types of the tested polymer after 7 days of culture. However, a significant increase in total protein was observed in all tested polymers cultured from 4 to 7 days (Figure 9). This finding, together with the viability results, suggested that PCL and SM-PCL were bioactive with less activation of cell proliferation.

### 3.11 Alkaline phosphatase (ALP) activity determination

**Figure 10.**
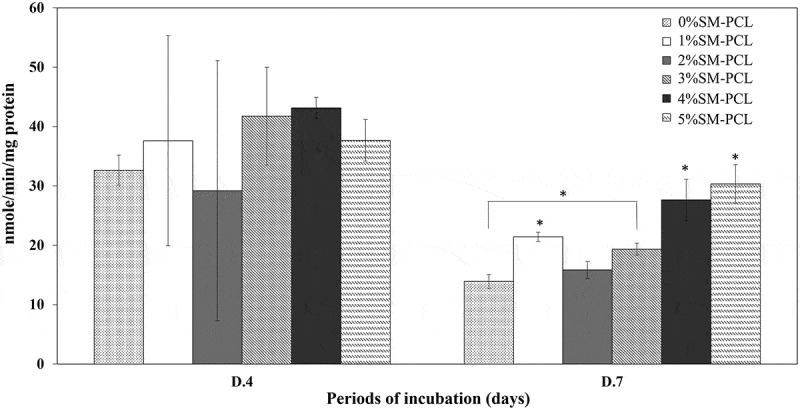
ALP activity of MG-63. ALP activity determination of MG-63 cells cultured with polycaprolactone (PCL) or sesamin-loaded PCL (SM-PCL) with concentrations of 1–5% (1%SM-PCL – 5%SM-PCL) for 4 (D.4) and 7 days (D.7). * indicates the significant difference among types of tested materials within the same period while # indicates the significant difference between two periods of incubation within the same types of the tested material with n =3; p < 0.05.

Sesamin significantly activated the ALP activity of MG-63 as a bone formation marker. The ALP activity was not significantly expressed in MG-63 cells cultured with PCL and SM-PCL at 4 days of investigation (Figure 10). The significant results were found in 7 days of a culture that 4%SM-PCL and 5%SM-PCL activities significantly enhanced the ALP activity in MG-63 cells. A slight reduction in ALP activity was observed from 4 to 7 days in all conditions. This finding suggested that SM-PCL are bioactive, especially with 4% and 5% sesamin, which positively affects bone formation.

## 4. Conclusions

In this study, sesamin-loaded PCL nanofiber membranes were successfully fabricated by electrospinning. The fiber diameter and entrapment efficiency of sesamin-loaded PCL membranes slightly increased with sesamin concentrations. The sesamin release mechanism was based on hydrolytic degradation of the polymers and involved a combination of chain cleavage and molecular weight loss. The kinetic release of sesamin from PCL nanofibrous membrane was the best-fitted with the Korsmeyer-Peppas, based on Fickian diffusion mechanism. The sesamin-loaded PCL nanofiber membranes showed biocompatibity indicating by cell attachment, viability, and proliferation of MG-63 cells. Moreover, the 4% and 5% SM-PCL nanofibrous membranes were bioactive and noticeable the positive effect on bone formation indicated by ALP bone maker expression. The combination of drug delivery and tissue engineering requires the scaffolds loaded with drugs to support the long-term release of drugs to aid the regeneration of damaged tissue. PCL loaded with sesamin showed promising membranes for drug delivery and bone applications in healthcare and biomedicine.
